# Keep me updated! Social support as a coping strategy to reduce the perceived threat caused by the cognitive availability of COVID-19 relevant information

**DOI:** 10.1007/s12144-021-01951-w

**Published:** 2021-06-16

**Authors:** Lisa Klümper, Svenja Sürth

**Affiliations:** 1 School of Human and Social Sciences, Social and Personality Psychology, Bergische Universität Wuppertal, Gaußstr. 20, 42119 Wuppertal, Germany; 2 Bergische Universität Wuppertal, Wuppertal, Germany

**Keywords:** COVID-19, Information availability, Perceived threat, Coping strategies, Seeking social support

## Abstract

The enormous amount of information about the COVID-19 pandemic in newspapers, TV channels, or social media reminds people every day of the potential threat the virus posed to their health and well-being in 2020. We examined if the cognitive availability of COVID-19 leads to the perception of heightened threat facilitating coping strategies and the moderating role of global self-efficacy and intolerance of uncertainty. A total of 235 participants randomly received either a newspaper article about the COVID-19 virus or Germany’s soil condition and were asked to indicate their current level of the perceived threat of the virus and the use of different coping strategies. Results indicate that the cognitive availability of COVID-19 information leads to a higher perceived threat, leading to more seeking for social support. Although neither self-efficacy nor intolerance of uncertainty moderates the effect of cognitive availability on the perceived threat, both personality characteristics moderated the relationship between perceived threat and different coping strategies. We discuss our results in line with current research on the impact of the COVID-19 pandemic on coping strategies and well-being.

## Introduction

Since early 2020, the novel virus SARS-CoV-2 (also known as COVID-19 or more colloquial Coronavirus) has spread worldwide. In December 2020, more than 70 million cases were confirmed, and over one million people died in the course of the virus, according to the World Health Organization (WHO, 2020). COVID-19 first appeared in Europe in February 2020 after spreading in Asia. In Germany, the Robert-Koch Institute (RKI) represents the government’s central scientific institution in the field of biomedicine and public health and is the most important scientific source of information about the COVID-19 pandemic. The RKI reveals information to the German population, and is frequently mentioned by the government and media. Following the RKI, it is difficult to make general statements about the disease progression because the course of COVID-19 is unspecific, diverse, and can vary significantly from individual to individual. The spectrum ranges from no symptoms to server pneumonia with lung failure that can end deadly. Pre-existing vulnerabilities, such as age and weight can influence a problematic course (RKI, 2020).

Consequently, almost every country ordered different public health strategies and protective measures to prevent the spread of the virus. In Germany, for example, people are constantly reminded of the health preventing AHA-strategy (“Abstand halten” = keep social/ physical distance, “Hände waschen” = wash your hands, “Alltagsmaske tragen” = wear a face mask). Also, during a national “lockdown” in Germany from March to April of 2020, all daycares and educational institutions, and all non-essential businesses, such as gyms and restaurants, were closed, and social contacts were restricted (for an overview and timeline, see Zacher & Rudolph, 2020).

Although social distancing and the restriction of social contacts are seen as a necessary intervention to prevent the spread of the virus, it threatens a basic human need to connect with other people (Baumeister & Leary, 1995). Loneliness, due to the perceived discrepancy between the actual and the desired quantity and quality of social relationships, has severe effects on the physical and mental health, well-being, and the general sense of safety (Stickley et al., 2016). As one public health intervention to prevent the spread of the COVID-19 virus, social distancing can aggravate feelings of loneliness and affect long-term health negatively (Van Bavel et al., 2020). Also, the social connection can help people regulate emotions, remain resilient, and social support is a crucial resource to cope with stress during difficult times (Jetten et al., 2012). During the COVID-19 pandemic, the threat caused by the virus can increase the perceived stress and the importance of social support as a coping strategy. If this relationship between the threat caused by the virus and seeking social support is empirically supported, the importance of discussing the government’s restrictions for social contacts would be highlighted. Therefore, the present study aimed to investigate the association between the availability of COVID-19 information, the perceived threat of COVID-19, and strategies of coping with the perceived threat.

### Perceived Threat and Increased Stress during the COVID-19 Pandemic

Different situations caused by physical or psychological factors can be perceived as internal or external threats leading to heightened feelings of stress (Braasch, 2018; Lecic-Tosevski et al., 2011). Following the classical transactional stress theory (Lazarus & Folkman, 1984), stress is defined as the discrepancy between the situation’s requirements and evaluating the available resources (Lazarus, 1991). According to the conservation resource model (CRM, Hobfoll, 1998), resources can be objective (e.g., clothes, houses, cars) or non-material (e.g., health, professional position), and personal resources (e.g., expertise, abilities). During a pandemic, several factors can lead to a loss of resources and, therefore, stress. For example, the potential loss of one’s own or loved one’s well-being and health, social isolation, confusion, and various fears of the future are all relevant stressors (Schnell & Krampe, 2020). Furthermore, the charge on the health care system and the devastating economic impact, which can threaten the personal economic resources, can lead to a higher stress perception (Callaway et al., 2020). Therefore, COVID-19 is perceived as a multidimensional, potential toxic stressor (Brakemeier et al., 2020), defined through five characteristics. First, it is a global phenomenon with an unpredictable duration leading to increased anxiety and uncertainty. Second, it has a systematic impact on several public life areas (e.g., the global and local economic system). Third, it impacts different personal life areas (e.g., family and social life, work-life). Fourth, it creates a subjectively perceived loss of control and helplessness (Röhr et al., 2020). Finally, COVID-19 and the related restrictions deny access to protective factors like free time activities or social contacts (Brakemeier et al., 2020; Gruber et al., 2020) that are essential resources according to the CRM model.

Recent studies indicate that the COVID-19 pandemic increases stress and stress-related symptoms (Duan & Zhu, 2020). For instance, people are concerned about their health and economic damage (Fetzer et al., 2020) and report higher frustration and isolation (Giallonardo et al., 2020). During the first wave of COVID-19 mental health issues have been raised (Rettie & Daniels, 2020). Significantly people who work in the health care system, like general practitioners, reported more helplessness and depressive symptoms (Amerio et al., 2020). Even though the pandemic itself is a significant source of stress, self-isolation policies can reinforce social isolation and relationship difficulties and increase individuals’ stress perception (Van Bavel et al., 2020). People experience more stressors, including health-related worries, job insecurity, and work-family conflicts (Blustein et al., 2020). These stressors can, in turn, affect mental well-being and health (see Brakemeier et al., 2020, for an overview).

Although the COVID-19 pandemic is a sustained stressor, several factors can temporarily heighten the perception of threat. In the case of the COVID-19 pandemic, today’s media coverage increases the availability of information about the virus and negative consequences extremely. The internet and social media represent a comparatively new way to disseminate information very quickly as the newest information is available at almost any time. This technological progress has positive effects but, for instance, can have an impact on the perceived risk, which in turn can lead to misjudgments (Werth et al., 2020). A potential underlying mechanism, which can lead to the experience of heightened perceived threat by COVID-19, might be the cognitive availability of COVID-19 information. As prior research showed, people overestimate the frequency and impact of an event depending on memory retrieval. This bias in information processing is called the availability heuristic (Tversky & Kahneman, 1973). For example, the availability heuristic may cause people’s overestimation of death’s probability through sensational causes on a victim (Slovic et al., 1980) and lead to a biased estimation of diseases (Agans & Shaffer, 1994). In the case of COVID-19, initial research indicates that the more time spent on tracking COVID-19 related news, the higher the individual level of state anxiety (Nekliudov et al., 2020). The subjectively perceived level of information is associated with an increase in COVID-19 related fear (Bäuerle et al., 2020). Moreover, Fetzer et al. (2020) found experimental evidence indicating that the perception and information regarding the mortality and contagiousness through the virus can influence individual expectations about the economy and personal situation during the COVID-19 pandemic significantly. These results lead to the assumption that the availability of COVID-19 related information can influence the individual perception of the threat by the virus.

### Coping with Perceived Threat during the COVID-19 Pandemic

As stated above, the perceived threat can lead to increased stress through the perceived discrepancy between the situation’s requirements and one’s resource, leading to several ways of coping with this stress. These coping strategies include cognitive and behavioral attempts to deal with the requirements resulting from the reciprocal relationship between a person and demanding environmental factors (Bossong, 1999). Based on Lazarus and Folkman (1984), the different coping strategies are either problem-solving or emotion-regulating. It is assumed that problem-solving strategies, compared to emotional-regulation strategies, are more efficient and more health-promoting (Schwarzer et al., 2004). However, the transactional theory neglects that humans are social beings and can solve problems and cope with stress in this context (Weber, 1997). The multiaxial coping model (Hobfoll, 1998, 2004), which is based on the conservation resources theory, addresses this social context and differentiates between the performance of active or passive strategies, which are either prosocial (e.g., seeking social support, considerate actions) or antisocial (e.g., aggressive actions) (Buchwald & Vogelskamp, 2007).

During the COVID-19 pandemic, recent studies highlighted the role of two coping strategies. Seeking social support is a coping strategy that positively includes other people in one’s problem-solving. Social support is defined differently but indicates the perception and experience that one is cared for, loved, and valued by others and part of a social network, including assistance and commitment (Wills, 1991). Perceived social support includes the appraisal of significant others’ availability to provide support, determining significant others’ as a social resource (Gottlieb & Bergen, 2010; Williams et al., 2004). The importance of social others and the social network is theoretically implied by the humans’ fundamental need to connect with others (Baumeister & Leary, 1995) and the idea that close social bonds are needed to deal with challenging life events from early childhood (Bowlby, 1969). According to Festinger’s social comparison theory (Festinger, 1954), which highlights the role of other people in times of insecurities, people want to eliminate subjective uncertainty about evaluating a situation and want to collect information about the appropriateness of their reactions by comparison with others. Such a social comparison occurs when there is no objective standard for a situation, as is the case within a global pandemic. For this purpose, the selection of a suitable comparison person is essential. Social comparison presupposes similarity as it is given in a reference group such as friends and family. In line with Festinger’s theory, Schachter (1959) proposed that people use other persons for self-evaluation, especially evaluating emotional reactions, and need to affiliate with others in stressful and fearful situations. Research has shown that people’s enhanced sadness by social loss increases the individual level of social connectedness and the desire to engage in social behavior (Gray et al., 2011).

In line with the predictions of social comparison theory, a stress-buffer hypothesis suggests that social support protects the individual from negative consequences of stress (e.g., worry or depressive thoughts) when confronting threatening life events (Cohen & McKay, 1984; Cohen & Wills, 1985). Whereas social isolation or exclusion increases the risk of physical and mental health issues (Moieni & Eisenberger, 2020), perceiving and receiving social support can protect from psychological problems (Bianco & Eklund, 2001; Cohen et al., 2018). Prior research indicates that perceived threat can increase the need for social support and the wish to exchange with others (Coelho et al., 2020). During the COVID-19 pandemic, research leads to first support for the stress-buffer hypothesis of social support. Results suggest that perceiving and receiving social support was associated with higher psychological health during the pandemic, whereas this was independent of worry about COVID-19 (Szkody et al., 2020). Buchwald and Begic (2020) found a positive correlation between perceived threat by COVID-19 and seeking social support, indicating that higher perceived threat leads to a higher seeking social support. Therefore, higher perceived threat during the COVID-19 pandemic will heighten the need for people to validate their feelings and thoughts with others and seek social support from friends and family to cope with stress.

Besides seeking social support, research indicates that the perceived threat by COVID-19 leads to a decrease of assertive actions as a coping strategy. Assertive actions can be described as the ability to persuade others and assert one’s own opinion. High assertive people perceived themselves as strong and dynamic and can rely on their own strength to solve problems. It is described as an active coping strategy that does not include others or the help from others to cope with the stressor in the problem-solving process (Braasch, 2018). During the COVID-19 pandemic, the reliance on one’s strength and abilities might be restricted due to resource loss and the heightened insecurities, which impact many parts of public and private life domains. First empirical evidence suggests a negative relationship between perceived threat by COVID-19 and the use of assertive actions as a coping strategy (Buchwald & Begic, 2020). Therefore, assertive actions as a coping strategy might be reduced with higher perceived threat by COVID-19.

### Impact of Individual Differences on Perceived Threat and Use of Coping Strategies

Besides the assumed connection between cognitive availability of COVID-19 relevant information, perceived threat, and social support and assertive actions as coping strategies, individual differences might influence these connections. According to Lazarus (1991), the reactions to threatening situations vary across individuals, and the response to a perceived threat can be unique and individual. That is why fear and worry are affecting psychological and physical health in varying degrees (Yendrembam et al., 2021). One factor that impacts perception is individual differences in personality traits, as traits can create differences in emotional, behavioral, and cognitive domains (McCrae & Costa, 1997). Prior research indicates that some people perceive more threats than others. For example, higher neuroticism levels predict the perception of events as threatening (Ebstrup et al., 2011) and lead to more amounts of perceived threat resulting from such an event (Lecic-Tosevski et al., 2011). In contrast, conscientiousness, optimism, or perceived control are associated with a more positive perception and reaction to threatening situations leading to more approach and problem-focused coping (Aspinwall & Brunhart, 2000; Ebstrup et al., 2011; Nes & Segerstrom, 2006).

In line with these findings, other individual dispositions impact the perception of the pandemic as a stressful event. For example, individual differences in life satisfaction and positive and negative affect were associated with different control-ability and threats (Zacher & Rudolph, 2020). Higher levels of emotionality (one facet of the HEXACO model of personality; Lee & Ashton, 2008) lead to a higher perceived threat by COVID-19, which predicts the amount of hoarding behavior (e.g., toilet paper stockpiling) during the pandemic (Garbe et al., 2020). Besides, the dark triad (narcissism, machiavellianism, and psychopathy) is negatively associated with health behavior and prevention, whereas it is positively related to the tendency to continue everyday life and hoarding behavior (Nowak et al., 2020; Triberti et al., 2021).

During the COVID-19 pandemic, individual differences in two dispositions appear to be related to a higher perceived threat. First, intolerance of uncertainty as a cognitive bias that influences perception, interpretation, and reaction to uncertain situations (Dugas et al., 1998) causes a specific reaction to threatening situations. It is a disposition to be upset by unknown aspects of a situation and moderates the perceived threat caused by an actual threat (Freeston et al., 2020). This reaction caused by uncertainty does not rely on the reasonable possibility of an event to occur (Hong & Lee, 2015). Intolerance of uncertainty is seen as a fundamental factor of worry (Freeston et al., 1994) and underlying many anxiety disorders (Morriss et al., 2016). For people high in intolerance of uncertainty, it is unacceptable that adverse events may occur, although such an event’s probability is minimal (Dugas et al., 2001). During the H1N1 pandemic, evidence supports this notion by showing that people with higher levels of intolerance of uncertainty reported more perceived threat and higher levels of anxiety than people with lower levels of intolerance of uncertainty (Taha et al., 2013). In the case of the COVID-19 pandemic, the unknown situation related to the virus might, therefore, especially impact people’s experience of perceived threat when they show higher levels of intolerance of uncertainty. First empirical evidence supports this link by showing that intolerance of uncertainty is associated with higher fear of COVID-19 and a higher emotional perception of the pandemic (Satici et al., 2020), whereas lower intolerance of uncertainty buffers the effects of social isolation on psychological distress (Smith et al., 2020). Therefore, intolerance of uncertainty may pose a risk factor for a heightened perceived threat. Besides this perception, higher levels of intolerance of uncertainty lead to a need for uncertainty-reducing behavior (Freeston et al., 2020). Thereby, intolerance of uncertainty appears to be determining in reactions to the perceived threat. During the H1N1 pandemic, for example, a higher intolerance of uncertainty was accompanied by a lower assessment of self and external control, which in turn goes along with a lower level of problem-focused and a higher level of emotionally-focused coping (Taha et al., 2013). Moreover, Rettie and Daniels (2020) have shown that people with a high intolerance of uncertainty are more likely to use maladaptive coping strategies, leading to psychological distress.

Besides intolerance of uncertainty, which may lead to a higher perceived threat and the need for more coping strategies to reduce this threat, individual differences in self-efficacy might positively impact the perceived threat and need for coping. Self-efficacy can be described as the belief to have the ability to achieve desired effects through one’s action, the belief of having the competency to cope with tasks or stressors, and influencing certain events (Bandura, 2006). As prior research indicates, self-efficacy can bias information processing, which can shape behavioral and emotional responses. Lower self-efficacy is associated with a higher negative reaction to threatening stimuli, whereas higher self-efficacy leads to a more adaptive reaction to threatening stimuli and facilitates well-being and health (Karademas et al., 2007). Previous research shows that self-efficacy is negatively related to higher levels of COVID-19 perceived risk (Yıldırım & Güler, 2020). Furthermore, initial evidence shows that an increased level of self-efficacy is accompanied by fewer COVID-19 specific threats and general health concerns (Buchwald & Begic, 2020). These results lead to the assumption that self-efficacy could be important for the perceived threat caused by COVID-19.

Also, self-efficacy is a part of a person’s skill set to cope with threats and might influence how to deal with the threat. Higher self-efficacy relates to more problem-solving coping strategies and more effective coping (Morales-Rodríguez & Pérez-Mármol, 2019). Karademas and Kalantzi-Azizi (2004) have shown that self-efficacy expectations are positively related to a positive approach and tension reduction coping strategies and, hence, negatively associated with psychological symptoms and self-isolation and passive acceptance coping strategies. Additionally, self-efficacy is associated with higher problem-solving but is negatively connected to seeking social support as an active, prosocial coping strategy (Li et al., 2018).

Therefore, intolerance of uncertainty and self-efficacy can, on the one hand, influence the perception of a threatening situation, and on the other hand, the use of coping strategies.

### The Present Research

In our present study, we want to examine the relationship between the cognitive availability of COVID-19 relevant information on the perceived threat caused by COVID-19 and the use of seeking social support and assertive action as coping strategies.

Several situations during the COVID-19 pandemic can remind people of threatening situations and the loss of their valuable resources increasing the perceived threat by the virus and the need for coping with this threat. As stated above, an essential factor influencing the amount of perceived threat by COVID-19 might be the confrontation with COVID-19 relevant information through newspapers, TV channels, or social media, leading to a heightened perceived threat posed by COVID-19 according to the availability heuristic. A few initial studies examined the relationship of information of COVID-19 on the perceived threat, using mostly correlative designs based on a retrospective report. We experimentally manipulated the cognitive availability of COVID-19 relevant information using newspaper articles to examine a possible causal relationship between exposure to information and the perceived threat. We assume that people who are confronted with COVID-19 relevant information would perceive more threat by COVID-19 than people in a control condition when no COVID-19 information is cognitively available.

As a consequence, the heightened perceived threat should impact the use of coping strategies. We focused primarily on two coping strategies, seeking social support and assertive actions, as prior research indicates their role during the COVID-19 pandemic. Recent research showed inconsistent results for the relationship of perceived threat by COVID-19 and seeking social support and only partly support the stress-buffer hypothesis (Buchwald & Begic, 2020; Szkody et al., 2020). To further examine the relationship between perceived threat and social support, we hypothesized that the heightened cognitive availability of COVID-19 information would lead to a more perceived threat, leading to a higher seeking for social support. Also, we tried to replicate Buchwald and Begic’s (2020) findings and, therefore, assumed that the higher perceived threat caused by higher cognitive availability of COVID-19 information leads to lower use of assertive action as a coping strategy.

Besides these assumed relationships between cognitive availability, perceived threat, and the two coping strategies, we consider individual differences in intolerance of uncertainty and self-efficacy that might influence an event’s perception as threatening and pose people at risk of a pandemic’s negative consequences for the well-being and mental health differently. Therefore, we hypothesized that the cognitive availability of COVID-19 relevant information leads to a higher perceived threat, especially for people with higher levels of intolerance of uncertainty. Due to this, we considered possible different needs for coping with the perceived threat and analyzed the impact of intolerance of uncertainty on the use of social support and assertive actions as a coping strategy. We assume that higher levels of intolerance of uncertainty lead to more seeking social support and fewer assertive actions. Also, we examined the impact of self-efficacy on the perception of threat and coping with this threat. Therefore, we hypothesized that lower self-efficacy leads to a stronger relationship between the cognitive availability of COVID-19 information and perceived threat and that higher levels of self-efficacy will lead to more assertive actions but lower seeking social support.

Besides seeking social support and assertive action as coping strategies, we explored the role of the perceived threat by COVID-19 and the impact on other coping strategies according to the multiaxial coping model to gain a greater insight into the impact of COVID-19 perceived threat and the need for several coping strategies. Therefore, we exploratively analyze the relationship between the perceived threat and other coping strategies suggested by the multiaxial coping model.

## Method

### Participants

Based on prior studies’ effect size on the relationship between perceived threat and coping strategies (Buchwald & Begic, 2020), an a priori sample size calculation with G*Power (Faul et al., 2009) was done. A minimum sample size of 212 participants was needed to detect the effect r = .19 (minimum power = .80; Alpha error probability = .05).

The recruitment of the participants was done via different social networks and different websites. A total of 238 participants took part in the experiment. Three persons had to be excluded because they expressed the right guesses about the aim of the study. The final sample included 235 participants (74.5% women). More than half of them were students (57.0%), and, therefore, half of the sample has a higher secondary school leaving certificate (“Abitur, Hochschulreife”). Age varied from 18 to 70 (*M* = 31.16; *SD* = 13.30). Participants in both experimental conditions reported similar experiences with the COVID-19 virus or protective measure (see Table 1).

**Table 1:** Participant’s experiences with a COVID-19 infection and related measures

	Cognitive Availability		Difference
	Experimental group COVID-19 (*n* = 121)^1^	Control group Soil condition (*n* = 114)^2^	χ^2^ (2, 235)
“I was tested positive for the Corona-Virus.”	1.70%	0.00%	χ^2^ = 2.28, *p* = .319
“I personally know someone who was tested positive for the Corona virus.”	35.50%	34.20%	χ^2^ = 0.35, *p* = .841
“In my hometown, there are proven cases of a Corona-Virus infection.”	82.60%	76.30%	χ^2^ = 1.19, *p* = .552
“I am currently in domestic isolation voluntarily”.	4.10%	6.10%	χ^2^ = 0.59, *p* = .781
“I was in domestic isolation voluntarily.”	34.70%	37.70%	χ^2^ = 0.72, *p* = .697
“I am currently in enforced quarantine because of an infection with the Corona-Virus.”	0.80%	0.90%	χ^2^ = 0.002, *p* = .961
“I was in enforced quarantine because of an infection with the Corona-Virus.”	5.00%	7.90%	χ^2^ = 0.82, *p* = .366
“I belong to a risk group.”	18.20%	10.50%	χ^2^ = 3.09, *p* = .213

*Note.* ‘Yes’ responses are shown in percentage; ^1^ 0.8–5.0% no responses; ^2^0.9–7.0% no responses

### Materials

#### Availability of COVID-19 Information

The availability of COVID-19 information was experimentally manipulated by presenting a newspaper article containing RKI’s information about COVID-19 to half of the participants. As the study was only conducted in Germany, the information was solely extracted from the website of the RKI. In contrast, the other half of the participants read a newspaper article from the German Federal Ministry of Food and Agriculture (2020) about the national soil condition. The control group was chosen because it has no relation to the pandemic and should not influence threat perception. Three items were included as a manipulation check to assess the newspaper articles’ credibility (1: *not at all credible* to 5: *very credible*) and difficulty to read (1: *very difficult to read* to 5: *very easy to read*) and to understand its content (1: *not at all* comprehensible to 5: *very comprehensible*) on a five-point scale. The experimental and control group perceived both articles as equally credible, *t*(214.06) = 1.78, *p* = .07, Cohen’s *d* = 0.97, 95% CI [−0.02, 0.49], but differ significantly on the difficulty of reading, *t*(199.13) = 5.60, *p* < .001, Cohens’s *d* = 1.01, 95% CI [0.47, 1.00], and comprehending the article, *t*(207.39) = 5.49, *p* < .001, Cohens’s *d* = 1.09, 95% CI [0.46, 0.99]. The COVID-19 article was more difficult to read and understand than the soil condition article (see Table 2). However, both the reading difficulty and comprehension difficulty were not associated with the perceived threat of COVID-19 (*p*s > .36).

**Table 2:** Descriptive statistics for the measurements depending on the experimental condition (COVID-19 vs. soil condition)

	Cognitive Availability			
	Experimental group COVID-19 (n = 121)		Control group Soil condition (*n* = 144)	
	*M*	*SD*	*M*	*SD*
Newspaper Article				
Credibility	4.03	0.86	3.81	1.08
Comprehensibility	4.54	0.82	3.79	1.19
Difficulty	4.17	0.92	3.39	1.25
Perceived Threat	2.58	0.54	2.40	0.61
Self-efficacy	2.87	0.43	2.89	0.43
Intolerance of Uncertainty	2.50	0.66	2.51	0.61
Coping Strategies				
Seeking social support	3.46	0.57	3.32	0.72
Assertive actions	3.46	0.59	3.40	0.59
Considerative actions	3.43	0.60	3.55	0.56
Indirect actions	2.53	0.77	2.45	0.72
Avoidance	2.15	0.68	2.27	0.56
Aggressive-antisocial actions	1.54	0.51	1.66	0.57
Instinctive actions	3.00	0.64	2.87	0.66

#### Perceived Threat by COVID-19

The perceived threat by COVID-19 was measured with an adapted German version of the Perceived Coronavirus Threat Questionnaire scale initially created by Conway et al. (2020). The German scale includes six items (e.g., “Thinking about the coronavirus (COVID-19) makes me feel threatened.”), which should be answered on a four-point scale and were summed up to a total perceived threat scale. The scale shows adequate internal consistency in the current German sample (α = .83), almost comparable to the reliability reported in prior research (Conway et al., 2020).

#### Coping Strategies

The 39 item version of the German stress coping inventory (Stressbewältigungsinventar, SBI; adopted from the Strategic Approach to Coping Scale; Schwarzer et al., 2003) was used to measure different coping strategies (see Table 2 for further information). Participants indicated how often they react in stressful situations with each type described by providing their agreement with several statements on a five-point scale (“Please indicate how often you react in the described manner in response to the stressful situation”). The main focus laid on the seeking social support subscale, which includes six items (e.g., “I check with friends about what they would do.”; α = .82), and the assertive action subscale (e.g., “I am assertive and forceful, but avoid harming others.”; α = .78), which describes the action to enforce one’s own will (Buchwald & Vogelskamp, 2007). The other statements represent the subscales aggressive-antisocial action (e.g., I seek for others’ weaknesses and use them for my advantage), indirect action (e.g., “I try to keep control but let others believe they are in authority”), instinctive action (e.g., “I rely on my instinct and not on my mind), considerate action (e.g.,” I am very very careful and consider all the alternatives”).

#### Self-Efficacy

The generalized self-efficacy scale (Schwarzer & Jerusalem, 1999) was used to measure self-efficacy. The scale consists of ten items (e.g., “I can always manage to solve difficult problems if I try hard enough.”), which should be answered on a four-point scale and were summed up to a total self-efficacy score. The scale shows adequate reliability in the sample (α = .87), comparable to prior reported reliabilities (e.g., Jerusalem & Schwarzer, 2003).

#### Intolerance of Uncertainty

The German version of the intolerance of uncertainty scale (UIS; Dietmaier et al., 2008) was used. The scale consists of 12 items (e.g., “My insecurity makes life unbearable”), which were answered on a five-point Likert Scale from 1 (“not at all”) to 5 (“exactly”). The UIS shows adequate reliability (α = .82), comparable to those reported in prior studies (e.g., Buhr & Dugas, 2002).

#### Information about Experiences with COVID-19

Participants had to indicate whether they were personally affected by the virus (being tested positive, being quarantined, being in isolation voluntarily, being a part of the risk group) or knew someone who was affected by the virus (knowing someone personally, knowing a person in the neighborhood) (see Table 1 for further information).

#### Demographic Information

In the end, participants should provide personal information about their sex, age, education, and current job. Participants could make free remarks and were asked about their guesses about the intention of the study.

### Procedure

The survey was created using an online survey tool (Leiner, 2019). Participants received a short instruction at the beginning of the study and were informed about the study’s course. The subjects were informed that the study aimed to examine the public opinion on Germany’s current topics, not to reveal the study’s real intention. After the participants agree with the privacy statement, they were randomly assigned to one of the two experimental conditions in which the newspaper article was presented (COVID-19 vs. soil condition article). After a given time frame of one minute, participants could continue the survey and received the perceived threat by COVID -19 scale. Then, they have to indicate different coping strategies. The self-efficacy and intolerance of uncertainty were presented in random order. At the end of the survey, participants indicate their experiences with the COVID-19 virus, their demographic data, and the newspaper articles’ comprehension. Participants did not receive compensation for participation and were free to contact the project supervisor for further questions.

### Data Analyses

All analyses were done using IBM SPSS Statistics 27. The SPSS macro PROCESS 3.5 was used for the analyses (Hayes, 2018). The experimental condition was contrast dummy-coded (1 = COVID-19 article, −1 = soil condition article). For the analyses, *p* values < .05 were reported as significant, while we used a Holm-Bonferroni correction for the *p* values (Holm, 1979). As we wanted to gain new insights into the dynamics of the pandemic, we reported findings with *p* values < .10 as marginally significant and discuss their relevance. For all analyses, the bootstrapped confidence interval (95%, 20,000 samples) will be presented.

## Results

For the descriptive statistics, internal consistencies, and inter-correlations of the measurements, see Table 2 and Table 3.

**Table 3:** Intercorrelations of the different measurements

	1.	2.	3.	4.	5.	6.	7.	8.	9.	10.
1. Perceived threat	(.83)									
2. Self-efficacy	−.13	(.87)								
3. Intolerance of uncertainty	.12	−.46^**^	(.82)							
4. Seeking social support	.29^**^	−.03	−.10	(.82)						
5. Assertive actions	−.08	.70^**^	−.43^**^	.01	(.78)					
6. Indirect actions	−.06	.06	.19^**^	−.15^*^	.16^*^	(.75)				
7. Instinctive actions	−.05	.11	−.13^*^	.04	.09	.10	(.80)			
8. Avoidance	−.06	−.36^**^	.34^**^	−.13	−.51^**^	.22^**^	−.21^**^	(.88)		
9. Aggresiv-antisocial actions	−.10	.01	.16^*^	−.23^**^	.07	.49^**^	.13^*^	.27^**^	(.78)	
10. Considerative actions	.19^**^	−.08	.21^**^	.29^**^	−.09	−.10	−.02	.17^**^	−.26^**^	(.64)

*Note*. * *p* < .05; ** *p* < .01; Cronbachs alpha is presented in the diagonal line in brackets

### Perceived Threat and the Use of Seeking Social Support and Assertive Actions

**Fig. 1:**
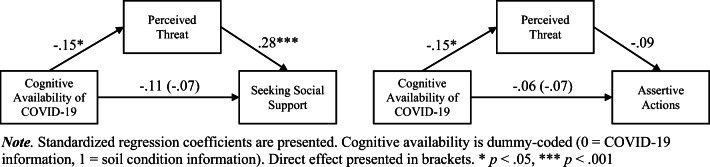
Results of the analyses of the relationship between cognitive availability of COVID-19 and the coping strategies seeking social support and assertive actions through perceived threat

As stated in the introduction, we assumed an impact of the cognitive availability of COVID-19 information on the amount of perceived threat, and, hence, a higher seeking social support and fewer assertive action. Therefore, in the first analysis, we used the experimental condition of cognitive availability of COVID-19 (COVID-19 vs. soil condition) as the dummy-coded independent variable, and the perceived threat by COVID-19 as the mediator. Seeking social support was the dependent variable. The analysis shows a significant effect of cognitive availability of COVID-19 relevant information so that people in the COVID-19 article condition perceived more threat, *b* = 0.09, SE = 0.04, *t*(233) = 2.31, *p* = .022, 95% CI [0.01,, 0.16], R^2^ = .023, *F*(1, 233) = 5.35. There was no significant direct effect of the cognitive availability on seeking social support when controlling for perceived threat, *b* = 0.04, SE = 0.04, *t*(232) = 1.04, *p* = .300, 95% CI [−0.04, 0.13], but higher perceived threat significantly lead to more seeking social support, *b* = 0.32, SE = 0.07, *t*(232) = 4.35, *p* < .001, 95% CI [0.17, 0.45], R^2^ = .088, *F*(2, 232) = 10.73. The total effect of cognitive availability, *b* = 0.07, *p* = .100, was reduced including the mediator, *b* = 0.04, *p* = .300, and the indirect effect of cognitive availability on seeking social support via perceived threat was significant, *b* = 0.03, SE = 0.01, 95% CI [0.004, 0.06]. The data supported the assumed relationship between cognitive availability of COVID-19 information, perceived threat and the coping strategy seeking social support (see Fig. 1, left panel).

Considering the role of assertive actions, we assumed a negative relationship between perceived threat and assertive actions as a coping strategy. Again, the cognitive availability of COVID-19 (COVID-19 vs. soil condition) was the dummy-coded independent variable, and the perceived threat by COVID-19 was the mediator. Assertive action was the dependent variable. The analysis showed a significant effect of cognitive availability of COVID-19 relevant information on the perceived threat, *b* = 0.09, SE = 0.04, *t*(233) = 2.31, *p* = .022, 95% CI [0.01, 0.16]. There was no significant effect of the cognitive availability on assertive action when controlling for perceived threat, *b* = −0.04, SE = 0.04, *t*(232) = 1.02, *p* = .308, 95% CI [−0.04, 0.13], and there was no significant effect of perceived threat on assertive actions, *b* = −0.09, SE = 0.07, *t*(232) = −1.22, *p* = .222, 95% CI [−0.23, 0.05]. Therefore, the assumed relationships did not fit the data (see Fig. 1, right panel).

### The Impact of Individual Differences on Perceived Threat and Coping Strategies

We assumed that individual differences in self-efficacy and intolerance of uncertainty would influence the stated relationships between cognitive availability of COVID-19 information, perceived threat, and seeking social support, and assertive action. Therefore, we included self-efficacy and intolerance of uncertainty as moderators of both the effect of cognitive availability of COVID-19 on the perceived threat and the effect of perceived threat on seeking social support and assertive actions as coping strategies.

Overall, there was no significant effect of self-efficacy, *b* = −0.12, SE = 0.11, *t*(229) = −1.08, *p* > .999, 95% CI [−0.35, 0.08], or intolerance of uncertainty, *b* = 0.07, SE = 0.08, *t*(229) = 0.994, *p* > .999, 95% CI [−0.07, 0.20], on perceived threat. Also, there was no significant interaction of self-efficacy × cognitive availability, *b* = 0.09, SE = 0.11, *t*(229) = 0.78, *p* = .436, 95% CI [−0.13, 0.30], or interaction of intolerance of uncertainty × cognitive availability, *b* = 0.02, SE = 0.07, *t*(229) = 0.32, *p* = .746, 95% CI [−0.11, 0.16], on the perceived threat, *R*^2^ = .046, *F*(5, 229) = 1.89, *p* = .097. Therefore, the data did not support our assumptions for the moderating role of individual differences on the amount of perceived threat by COVID-19.

Considering the prediction of seeking social support, *R*^2^ = .139, *F*(6, 228) = 5.23, *p* < .001, higher intolerance of uncertainty marginally predicts higher seeking social support, *b* = −0.17, SE = 0.07, *t*(228) = −2.51, *p* = .052, 95% CI [−0.31, −0.04], whereas this was qualified by a significant perceived threat × intolerance of uncertainty interaction, *b* = 0.34, SE = 0.13, *t*(228) = 2.65, *p* = .045, 95% CI [0.10, 0.60], Δ*R*^2^ = .03, *F*(1, 228) = 7.02, *p* = .008. Simple slope analysis revealed a significant effect for intermediate (50th percentil), *b* = −0.29, SE = 0.07, *t*(229) = 3.39, *p* < .001, 95% CI [0.15, 0.43], and high values (84th percentil), *b* = 0.49, SE = 0.09, *t*(230) = 5.18, *p* < .001, 95% CI [0.30, 0.68], of intolerance for uncertainty. There was no significant effect for low levels (16th percentil) of intolerance of uncertainty, *b* = −0.67, SE = 0.12, *t*(230) = 1.10, *p* = .272, 95% CI [−0.09, 0.32]. The relationship between cognitive availability, higher pereceived threat and more seeking social support was only significant for intermediate, *b* = 0.03 SE = 0.01, 95% CI [0.003, 0.05], and high levels of intolerance of uncertainty, *b *= 0.04 SE = 0.03, 95% CI [0.01, 0.08]. Self-efficacy had no effect on seeking social support, *b* = −0.12, SE = 0.11, *t*(228) = −1.12, *p* = .817, 95% CI [−0.33, 0.09], and there was no self-efficacy × perceived threat interaction, *b* = 0.10, SE = 0.16, *t*(228) = 0.63 *p* = .801 95% CI [−0.21, 0.42].

Considering the relationship between perceived threat and assertive actions, results indicated that higher self-efficacy predicts higher use of assertive actions as a coping stratgy, *b* = 0.90, SE = 0.07, *t*(228) = 12.75, *p* < .001, 95% CI [0.76, 1.04], whereas there was no significant interaction of self-efficacy and perceived threat, *b* = 0.21, SE = 0.11, *t*(229) = 1.88, *p* = .244, 95% CI [−0.01, 0.41]. Also there was no significant effect of intolerance of uncertainty on assertive action, *b* = −0.11, SE = 0.05, *t*(228) = −2.31, *p* = .110, 95% CI [−0.20,0.02], nor a significant interaction of intolerance of uncertainty and perceived threat, *b* = 0.17, SE = 0.10, *t*(228) = 1.62, *p* = .322, 95% CI [−0.03, 0.35].

Overall the results only partly support our assumptions about the impact of individual differences perceived threat and coping strategies.

### Effects of the Perceived Threat and Individual Differences on Other Coping Strategies

We conducted additional exploratory moderator analyses to explore possible relationships of perceived threat on the other coping strategies besides seeking social support and assertive action. Therefore, the perceived threat served as the independent variables, self-efficacy, and intolerance of uncertainty were moderators (mean-centered), and the five coping strategies were the dependent variable. The experimental condition was included as a covariate (see Table 4).

For the coping strategy indirect actions, perceived threat did not predict the indirect action directly, *b* = −0.09, SE = 0.09, *t*(228) = −0.99, *p* = .771, 95% CI [−0.25, 0.08]. Higher intolerance of uncertainty lead to more indirect actions as a coping stratgy, *b* = 0.34, SE = 0.09, *t*(228) = 3.81, *p* < .001, 95% CI [0.17, 0.51], and this effect was qualified by an intolerance of uncertainty × perceived threat interaction, *b* = −0.42, SE = 0.16, *t*(228) = −2.59, *p* = .050, 95% CI [−0.73, −0.12], Δ*R*^*2*^ = .028, *F*(1, 228) = 6.72, *p* = .050. Simple slope analysis of the interaction showed a significant effect of perceived threat on indirect actions only for high levels (84th percentil) of intolerance of uncertainty, *b* = −0.29, SE = 0.10, *t*(229) = −2.79, *p* = .006, 95% CI [−0.49, −0.09]. There was no effect for intermediate (50th percentil), *b* = −0.06, SE = 0.09, *t*(229) = −0.65, *p* = .514, 95% CI [−0.24, 0.12], or low levels, (16th percentil), *b* = 0.14, SE = 0.14, *t*(229) = 0.99, *p* = .322, 95% CI [−0.14, 0.42], of intolerance of uncertainty.

Also, higher perceived threat, *b* = 0.19, SE = 0.07, *t*(228) = 2.69, *p* = .040, 95% CI [0.06, 0.32], and higher intolerance of uncertainty lead to more considerate action, *b* = 0.20, SE = 0.07, *t*(228) = 2.86, *p* = .036, 95% CI [0.06, 0.33], whereas there was no significant interactions.

There were no more significant effects of perceived threat, self-efficacy, or intolerance of uncertainty on the coping strategies (see Table 4).

**Table 4:** Results of the moderation analyses of self-efficacy and intolerance of uncertainty on the relationship between perceived threat and different coping strategies

		*b*	SE	*t*	*p*	95% CI	
Indirect Actions						Lower	Upper
Constant		2.50	0.05	52.13	< .001	2.41	2.59
Perceived Threat		−0.09	0.09	−0.99	.771	−0.25	0.08
Intolerance of Uncertainty		0.34	0.09	3.81	<.001	0.17	0.51
Perceived Threat × Intolerance of Uncertainty		−0.42	0.16	−2.59	.050	−0.73	−0.12
Self-efficacy		0.32	0.15	2.12	.140	0.05	0.63
Perceived Threat × Self-efficacy		−0.18	0.27	−0.68	.771	−0.70	0.29
Experimental Condition		0.06	0.05	1.14	.771	−0.04	0.15
		*R*^2^ = .105, *F*(6, 228) = 4.00, *p* = .001 Δ*R*^2^ = .028, *F*(1, 228) = 6.72, *p* = .050					
Avoidance							
Constant		2.21	0.04	56.22	< .001	2.14	2.29
Perceived Threat		−0.12	0.07	−1.88	.328	−0.26	0.01
Intolerance of Uncertainty		0.22	0.07	3.29	.005	0.09	0.35
Perceived Threat × Intolerance of Uncertainty		−0.22	0.14	−1.63	.330	−0.46	0.06
Self-Efficacy		−0.44	0.10	−4.25	< .001	−0.65	−0.25
Perceived Threat × Self-efficacy		−0.29	0.19	−158	.330	−0.63	0.09
Experimental Condition		−0.04	0.04	−1.10	.330	−0.12	0.03
		*R*^2^ = .196, *F*(6, 228) = 8.72, *p* < .001					
Aggressive-antisocial actions							
Constant		1.60	0.04	44.48	< .001	1.54	1.68
Perceived Threat		−0.08	0.06	−1.64	.510	−0.20	0.03
Intolerance of Uncertainty		0.18	0.07	2.69	.048	0.06	0.31
Perceived Threat × Intolerance of Uncertainty		−0.16	0.12	−1.31	.576	−0.38	0.06
Self-Efficacy		0.13	0.10	1.23	.576	−0.06	0.32
Perceived Threat × Self-efficacy		0.004	0.17	−0.05	.963	−0.31	0.30
Experimental Condition		−0.05	0.04	−1.48	.564	−0.12	0.02
		*R*^2^ = .066, *F*(6, 228) = 2.32, *p* = .034					
Considerate actions							
Constant		3.49	0.04	94.19	< .001	3.42	3.57
Perceived Threat		0.19	0.07	2.69	.040	0.06	0.32
Intolerance of Uncertainty		0.20	0.07	2.86	.036	0.06	0.33
Perceived Threat × Intolerance of Uncertainty		0.10	0.14	0.70	> .999	−0.15	0.37
Self-Efficacy		0.07	0.12	0.57	> .999	−0.16	0.28
Perceived Threat × Self-efficacy		0.17	0.22	0.78	> .999	−0.22	0.57
Experimental Condition		−0.08	0.04	−1.98	.196	−0.15	−0.004
		*R*^2^ = .094, *F*(5, 228) = 2.89, *p* = .010					
Instinctive Actions							
Constant		2.92	0.04	68.33	< .001	2.84	3.01
Perceived Threat		−0.05	0.07	−0.71	.477	−0.19	0.08
Intolerance of Uncertainty		−0.10	0.07	−1.32	.189	−0.24	0.05
Perceived Threat × Intolerance of Uncertainty		0.01	0.13	0.05	.960	−0.24	0.26
Self-Efficacy		0.10	0.13	0.78	.439	−0.15	0.37
Perceived Threat × Self-efficacy		0.08	0.17	0.44	.660	−0.26	0.42
Experimental Condition		0.05	0.04	1.22	.224	−0.03	0.14
		*R*^2^ = .030, *F*(6, 228) = 0.98, *p* = .437					

*Note*. Experimental condition was included as a covariate. CI = 95% Bootstrap confidence intervals (20,000 samples) are presented. Holm-Bonferroni adjusted *p* values are presented. Change in *R*^2^ is only presented in case of significant moderator effects

## Discussion

In the current study, we examined the effect of the cognitive availability of COVID-19 information as a cause of heightened perceived threat by COVID-19. Also, we tried to clarify the use of seeking social support and assertive actions as coping strategies due to this perceived threat. Finally, we wanted to examine the impact of individual differences in self-efficacy and intolerance of uncertainty on the perception of threat and the use of different coping strategies.

Therefore, we experimentally manipulated the cognitive availability of COVID-19 by confronting half of our participants with a newspaper article including information about COVID-19, whereas the other half of the participants received a newspaper article being not related to COVID-19. All participants should indicate the perceived threat and the use of different coping strategies. We hypothesized that the cognitive availability of COVID-19 information leads to a higher perceived threat, leading to higher seeking social support and lower levels of assertive actions. Our results can partly support these hypotheses. When the COVID-19 information is cognitively available, participants reported more perceived threat by COVID-19, which leads to more seeking social support as a coping strategy. However, there was no effect on assertive actions as a coping strategy.

We hypothesized that differences in self-efficacy and intolerance of uncertainty would moderate the relationship of cognitive availability on the perceived threat. These hypotheses are not compatible with our data. These personality characteristics do not moderate threat experiences. However, we found a moderation of the relationship between the perceived threat and different coping strategies so that higher levels of intolerance of uncertainty lead to a stronger relationship between perceived threat and seeking social support.

Considering the other coping strategies besides seeking social support and assertive action, higher levels of intolerance of uncertainty lead to a greater relationship between perceived threat and indirect actions as a coping strategy. Finally, perceived threat leads to more considerate actions, whereas this effect was independent of personality characteristics.

Overall, our findings support the multiaxial coping model (Hobfall, 1998, 2004) and point out that human perception and behavior in crises, such as a global pandemic, should be seen in the social context as seeking social support is an essential coping strategy. In line with prior research, our results support the link between perceived threat and seeking social support (Buchwald & Begic, 2020), and this relationship is more substantial depending on individual personality characteristics. The findings support the stress-buffer hypothesis partly, as there is no direct relationship between cognitive availability of COVID-19 information on seeking social support but only mediated through the perceived threat. Furthermore, this result goes along with Festinger’s social comparison theory (Festinger, 1954) since participants, who felt threatened by the availability of COVID-19 information, are more likely to seek social support to validate their perception and reaction to figure out how to behave during the pandemic. This leads to concerns about the government’s restriction on social contacts and family gatherings. During the lockdown in Germany, the government restricted private meetings to two households and a maximum of five persons. The government also recommended having no unnecessary social contact and living in self-isolation to stop spreading the virus. However, the restricted possibility of seeking social support may hinder people from coping with their stress, which leads to lower levels of well-being and mental health issues. Both the perception and reception of social support from close friends, family, or co-workers can positively impact mental health and well-being (Szkody et al., 2020). The health prevention strategies and the restrictions of social contacts could complicate social support’s perception and reception. For example, social contacts are especially relevant to a teenager’s personal and social development (Bzdok & Dunbar, 2020). Although real social encounters can be compensated for by communicating through different online channels, it can only partly replace real social activities (Vlahovic et al., 2012). Also, it might be possible that the decrease of real social encounters is more adverse for one group of people than for others. For example, several new ways to stay in contact with friends and family via videocalls or text messages pose challenges to older people or people who do not possess the needed hard or software. Our results highlight the importance of social contacts during a global pandemic and the need to implement ways to stay connected safely.

Another important result is the cognitive availability’s influence on the perceived threat posed by COVID-19, which is in line with prior research. When people spend an increasing time consuming COVID-19 relevant news, they experience increasing anxiety (Bäuerle et al., 2020; Nekliudov et al., 2020). Although prior research found such relationships, they are correlative by nature. Our experimental manipulation of the cognitive availability posed a method to investigate a potential causal relationship and provides further evidence for a potential underlying mechanism leading to threat and increases the risk of potential psychological harm. The processes described through the availability heuristic (Werth et al., 2020) seem to influence the perceived threat by COVID-19. This relationship is especially pronounced when people are highly intolerant of uncertainty and worry about potential negative outcomes. People are confronted with much information about COVID-19 and the current situation in their federal state or town and can receive new information almost every minute by different social media platforms, TV channels, and internet websites. It is essential to be informed about the virus and its consequences and prevent the spread of the virus. As recent research shows, higher information-seeking behavior leads directly and indirectly through worry to more preventing behavior (at least for people in China, Liu, 2020). However, our results indicate that people should not continuously consume information about the virus, which increased the perceived threat and can lead to constant stress experiences. This stress might decrease psychological well-being (Duan & Zhu, 2020; Xiong et al., 2020). Our results highlight the importance of using different information and being aware of the potential impacts of confrontation with this information.

Furthermore, the previously presented results indicate that individual differences in people’s reactions to uncertainties are crucial for the threat experience and influence how people react to a pandemic. This is consistent with the findings of Taha et al. (2013), which were able to show similar connections during the H1N1 pandemic. Moreover, the basic assumption that the construct is a cognitive bias that negatively influences the perception, interpretation, and response to unsafe situations (Dugas et al., 1998) can also be supported by our results. The pandemic put people at risk differently, not only through the virus’s physical harm but also through its economic and social life consequences. Our results highlight the importance of supporting people according to their vulnerabilities (e.g., a disposition to worry a lot) and strengthening potential resources (e.g., increase beliefs in one’s abilities to cope with challenging life events).

Our result showed that the perceived threat leads to more considerate actions, independent of differences in personality characteristics. Considerate actions are a passive, prosocial coping strategy and include considering others’ well-being and acting cautiously while considering all possible impacts on the others (Braasch, 2018). These actions are essential during a pandemic, as adherence to health prevention strategies like the AHA-rule is crucial to prevent harming others’ well-being. Some people do not adhere to health prevention strategies (e.g., wearing a mask) and do not adhere to the government’s restrictions (e.g., organizing huge illegal social gatherings) or protest against these restrictions. These actions are not considerate, and, based upon our results, it might be possible that these people either do not perceive threat by COVID-19 or cope differently with this perceived threat. As mentioned in the introduction, recent results suggest that higher values on the dark triad lead to fewer health prevention strategies (Nowak et al., 2020; Triberti et al., 2021). It would be interesting whether differences in the dark triad or other more adverse personality traits lead to fewer perceived threats or different coping with the threat.

### Limitations and Future Directions

We experimentally manipulated the cognitive availability of COVID-19 information by randomly presenting either a newspaper article about COVID-19 information or the soil condition in Germany to the participants. As reported in the method section, our two experimental groups show significant differences in the easiness of reading and comprehend the articles, although there was no significant difference in the credibility between the groups. Although the reading and comprehension difficulty has no statistical effect on the perceived threat, future research should consider these differences. Therefore, one should create newspaper articles that are equally difficult to read and comprehend.

In line with this critic, we have to acknowledge that we did not include a test for our experimental manipulation. As this is lacking in our study, we can not clearly state whether the effects are due to the cognitive availability of COVID-19 information or not. A test for the manipulation should be included in future studies to support our stated hypotheses further. In social psychology, an issue occurs using manipulation checks because they may impact participants’ awareness and suspicion of the relevant construct being studied (Hauser et al., 2018). However, this issue calls for the implementation of pilot validation of the manipulation. Therefore, in future investigations, the manipulation’s validity to the relevant underlying effect should be examined in a pilot study (Chester & Lasko, 2021).

We could not examine the effects of the relationships of cognitive availability, perceived threat, and coping strategies on our participants’ psychological health or well-being as we did not include measurements of these constructs. However, it would be interesting to explore the effects on well-being and thoroughly test the stress-buffer hypothesis. Our results only suggest that higher threat lead to the need to cope with stress through the social network but can not tell if this coping lead to an adaptive coping with stress, and fewer negative impacts on well-being and mental health. As prior research indicates, the COVID-19 pandemic is negatively related to well-being, and people report an increased level of depressive symptoms and anxiety disorders. The negative impact of COVID-19 and mental health is somewhat especially pronounced for subgroups of people. As mentioned, for example, general practitioners reported severe depressive symptoms and helplessness during the COVID-19 pandemic (Amerio et al., 2020). The current situation due to COVID-19 may also increase pre-existing psychological disorders (e.g., eating disorders, Rodgers et al., 2020). For example, prior research indicates that depressed individuals show a unique sensory processing pattern (Serafini et al., 2017), which could also be a crucial factor in the pandemic. As the hypo-and hypersensitivity to stimuli may be relevant factors in the emergence of affective disorders, it would be important to include these processing patterns in the context of reactions to threatening stimuli during the COVID-19 pandemic. These processing patterns may exaggerate adverse emotional reactions, leading to a higher reactivity to potentially threatening stimuli (Serafini et al., 2017).

Future research should also examine the effects of a mismatch between seeking social support and the real possibility of receiving social support. As stated above, the government’s restrictions on social gatherings, social distancing, limitation of contact, and the need for self-isolation may interfere with the need for social support. Furthermore, social support may have changed during the COVID-19 pandemic. It is more difficult to see each other in person during the pandemic, and social contact takes place predominantly online. Direct forms of social support might be hindered, which leads to a more indirect form of social support. In this context, future research could also investigate whether seeking social support “online” compared to seeking social support “offline” can function equally well as a coping strategy and whether there are age differences or not.

### Conclusion

The COVID-19 pandemic poses different challenges to the health care system, the government, and the economic system. Some people are more at risk of the virus, and people are differently affected by economic constraints. Also, people suffer differently from the restrictions and health prevention strategies, as some rely more on their social network to cope with stress than others.

## Data Availability

All data and the used material will be available in the Open Science Framework (https://osf.io/yw643/?view_only=b178de3ddf93405590849c0e6c6cd0a6).
